# Diagnostic Accuracy of 12‐Lead ECG Algorithms for Accessory Pathway Localization in WPW: A Meta‐Analysis

**DOI:** 10.1111/pace.70387

**Published:** 2026-08-11

**Authors:** Elsayed S. Moubarak, Ahmed Farid Gadelmawla, Almothana Manasrah, Anas Mansour, Mohamed S. Elgendy, Omar Elkoumi, Husam Abu Suilik, Suhaib El Khatib, Naser Albekaen, Ahmed Moustafa, Rania Samir, Islam Saboukh, Nadica Trajkovska, Angeliki Darma, Vanessa Sciacca, Thomas Fink, Moneeb Khalaph, Christian Sohns, Philipp Sommer, Hassan El‐Shirbiny

**Affiliations:** ^1^ Faculty of Medicine Cairo University Cairo Egypt; ^2^ Faculty of Medicine Menoufia University Menoufia Egypt; ^3^ Department of Internal Medicine United Health Services—Wilson Medical Center Johnson City New York USA; ^4^ Faculty of Medicine Al‐Azhar University Cairo Egypt; ^5^ Faculty of Medicine Tanta University Tanta Egypt; ^6^ Faculty of Medicine Suez University Suez Egypt; ^7^ Faculty of Medicine The Hashemite University Zarqa Jordan; ^8^ Department of Internal Medicine UCHealth Parkview Medical Center Pueblo Colorado USA; ^9^ Department of Internal Medicine Prince Hamza Hospital Amman Jordan; ^10^ Department of Cardiology, Faculty of Medicine Ain Shams University Cairo Egypt; ^11^ Cardiology Department, Faculty of Medicine Kafr Elsheikh University Kafr El Sheikh Egypt; ^12^ Department of Electrophysiology, Heart and Diabetes Center NRW, Ruhr University Bochum Bad Oeynhausen Germany

**Keywords:** accessory pathway, catheter ablation, diagnostic accuracy, electrophysiological study, meta‐analysis, systematic review, ventricular pre‐excitation, Wolff–Parkinson–White syndrome

## Abstract

**Background:**

In Wolff–Parkinson–White (WPW) syndrome, accurate localization of accessory pathways before catheter ablation can improve procedural planning and efficiency. Several 12‐lead electrocardiographic (ECG) algorithms have been proposed, but their diagnostic performance has not been consistently established. We performed a systematic review and meta‐analysis to assess the accuracy of ECG‐based algorithms for accessory pathway localization in manifest WPW.

**Methods:**

Following PRISMA‐DTA recommendations, PubMed, EMBASE, Cochrane CENTRAL, Web of Science, and Scopus were searched from January 1990 to January 2026. Studies enrolling adult or pediatric patients with manifest pre‐excitation who underwent electrophysiological study (EPS) and catheter ablation were included. Successful localization during EPS/ablation served as the reference standard. Random‐effects models were used to calculate pooled diagnostic accuracy, sensitivity, specificity, and diagnostic odds ratios (DOR). Summary receiver operating characteristic and leave‐one‐out sensitivity analyses were also performed.

**Results:**

Seventeen studies involving 3159 patients met inclusion criteria. The pooled diagnostic accuracy of ECG algorithms was 0.91 (95% CI 0.85–0.94), although heterogeneity was substantial (I^2^ = 91.4%). Pooled sensitivity was 0.87 (95% CI 0.73–0.94), and pooled specificity was 0.98 (95% CI 0.95–0.99; I^2^ = 0%). The pooled DOR was 297.17 (95% CI 46.33–1906.19; *p*<0.0001). Sensitivity analyses showed stable results after sequential exclusion of individual studies.

**Conclusion:**

Twelve‐lead ECG algorithms provide high diagnostic performance for accessory pathway localization in manifest WPW and may be useful adjuncts for pre‐ablation planning, although invasive electrophysiological mapping remains essential.

## 1 Introduction

Wolff–Parkinson–White syndrome (WPW) is a congenital condition that was first described in 1930, characterized by the presence of accessory pathway that connects the atria and ventricles, bypassing the normal AV conduction [1]. The anterograde conduction through accessory pathway causes ventricular pre‐excitation that manifests on ECG as a short PR interval and a delta wave.

In the majority of patients, WPW remains asymptomatic, a condition that is commonly known as the WPW pattern, while in others, it is associated with symptoms like palpitations, syncope, arrhythmias, and sudden cardiac death [2, 3, 4].

Catheter ablation using cryoablation and radiofrequency ablation has emerged as the standard treatment for WPW syndrome [5, 6, 7]. However, the safety, efficacy, and procedural success of either technique depend on accurate pre‐procedural localization of the accessory pathway.

ECG remains the primary non‐invasive tool for localization of the AP, and numerous ECG‐based algorithms have been developed for this purpose. Some algorithms focused on delta‐wave morphology [8] and QRS polarity [9], while others incorporated more detailed anatomical localization and multistep criteria [10, 11]. Despite their widespread use, these algorithms differ significantly in their complexity, anatomical definitions, and accuracy in localizing the AP.

This heterogeneity presents a challenge for clinicians when selecting the optimal method to localize the AP. To address this gap, we conducted a systematic review and meta‐analysis of 17 studies to determine the diagnostic accuracy, sensitivity, and specificity of these algorithms for localizing accessory pathways in patients with WPW syndrome.

## 2 Methodology

We conducted a systematic review and meta‐analysis of diagnostic test accuracy studies to synthesize evidence from studies comparing different surface ECG algorithms for localizing accessory pathways in WPW. This review followed the Preferred Reporting Items for Systematic Reviews and Meta‐Analysis of Diagnostic Test Accuracy Studies (PRISMA‐DTA) (Table S1) [12].

### 2.1 Protocol Registration

The review protocol was developed in accordance with the Cochrane Handbook for Systematic Reviews of Diagnostic Test Accuracy and the PRISMA 2020 statement [13]. It was registered in PROSPERO (CRD420261390465).

### 2.2 Data Sources & Search Strategy

We comprehensively searched electronic databases, including PubMed, EMBASE, the Cochrane Central Register of Controlled Trials, Web of Science, and SCOPUS. The search strategy was constructed to capture studies evaluating ECG algorithms for accessory pathway localization by using combinations of terms such as Wolff–Parkinson–White, WPW, preexcitation, and accessory pathway. These were combined with terms related to the index tests and outcomes, including algorithm, criteria, score, localization, sensitivity, specificity, accuracy, and validity published between January 1, 1990 and January 1, 2026. The search strategy was developed and adapted for each database (Table S2).

### 2.3 Study Selection

Two review authors independently screened article titles, abstracts, and full texts to identify eligible studies. Studies were included if they evaluated adult and pediatric patients with manifest WPW, defined by a visible delta wave, who underwent an electrophysiology study and radiofrequency ablation. Eligible studies evaluated any specific surface ECG algorithm designed to localize the accessory pathway, such as SMART‐WPW, EASY‐WPW, Pambrun, or Arruda. For all analyses, the reference standard was the accessory pathway location established during EPS and confirmed by the site of successful ablation. When an included study applied more than one ECG localization algorithm to the same cohort, each algorithm was extracted as a separate index‐test evaluation; those algorithms were not treated as reference standards or diagnostic comparators.

Included studies reported primary overall diagnostic accuracy, expressed as the percentage of correctly predicted sites, or secondary metrics including sensitivity and specificity for specific anatomical zones. Eligible study designs included observational studies (retrospective and prospective cohort studies) and randomized trials. We excluded studies involving patients with concealed pathways lacking a delta wave, those with structural heart disease such as Ebstein's anomaly, cases of pacing‐induced pre‐excitation, and failed ablation procedures lacking a confirmed gold standard location. Case reports and review articles were also excluded.

### 2.4 Data Collection, Data Extraction, Risk of Bias, and Applicability

Data were extracted using a predefined spreadsheet by one reviewer and independently verified by a second reviewer. We extracted two‐by‐two classification tables including true positives, false positives, false negatives, and true negatives, alongside sensitivity and specificity estimates. We also extracted the ECG algorithm and study characteristics, as detailed in Table 1. To ensure comparability across varied nomenclature in the primary literature, target anatomical regions were standardized into four core categories. The left free wall category included left lateral, left anterolateral, left posterolateral, and mitral annulus pathways. The right free wall category consisted of right lateral, right anterolateral, right posterolateral, and tricuspid annulus pathways. Septal pathways encompassed anteroseptal, midseptal, posteroseptal, para‐Hisian, right septal, and left septal locations. Finally, the coronary sinus category included the middle cardiac vein, the coronary sinus diverticulum, and the epicardial pathways. Studies that did not separately report coronary sinus locations were grouped with the septal or posteroseptal categories. Methodological quality was assessed using the Quality Assessment of Diagnostic Accuracy Studies tool (QUADAS‐2) [14], evaluating patient selection, the index test, the reference standard, and flow and timing. Disagreements in study selection, data extraction, or methodological quality assessment were settled through discussion or by consulting a third review author as an arbitrator.

**TABLE 1:** Summary characteristics of the included trials.

								Inclusion criteria							
Study ID	Study design	Blinding	Country	No of centers	Total patients	Algorithm (Index test)	ECG features used	Age group	Target condition	ECG rhythm	Accessory pathway	Medication/washout	Prior ablation	Reference‐standard	Primary outcome
Khalaph et al. 2025 [20]	Retrospective derivation + prospective validation	No	Germany + Japan	Multicenter	260	SMART‐WPW algorithm	Delta‐wave polarity QRS/delta polarity	Both pediatric & adult	WPW syndrome	NSR	Single AP	3 half‐lives before ablation	Excluded	EPS with successful RF ablation	Accurate localization of AP
Neto et al. 2024 [24]	Retrospective validation study	Yes	Brazil	Single‐center	138	EPM algorithm	Delta‐wave polarity QRS transition	Adults ≥18 years	Ventricular pre‐excitation or WPW syndrome	—	Single AP	—	—	Radioscopic position of the AP	Average diagnostic accuracy
El‐Hamriti et al. 2022 [21]	Retrospective derivation + prospective validation	Yes	Germany	Multicenter	211	EASY‐WPW algorithm	Delta‐wave polarity QRS transition	Both pediatric & adult	WPW syndrome	—	Single AP	3 half‐lives before ablation	Included	EPS with successful RF ablation	Exact localization of AP
Raharjo et al. 2022 [26]	Cross‐sectional + retrospective	Yes	Indonesia	Single‐center	47	Simple ECG Algorithm (2‐step)	QRS polarity QRS transition	Adults ≥18 years	WPW syndrome	—	—	—	Excluded	EPS with successful RF ablation	Diagnostic performance and inter‐observer agreement
Madadi et al. 2020 [23]	Cross‐sectional + retrospective	Yes	Iran	Single‐center	294	Right‐sided ECG algorithm (4‐step, polarity‐sum based)	Delta‐wave polarity QRS polarity	Both pediatric & adult	WPW syndrome	NSR or AVRT	Right‐sided AP	—	—	EPS with successful RF ablation	Ability of ECG algorithm to differentiate 8 right‐sided pathway locations
Baek et al. 2020 [16]	Retrospective derivation + prospective validation	—	South Korea	Two‐centers	262	New pediatric ECG localization algorithm — septal screening	Delta‐wave polarity QRS polarity QRS transition	Pediatric ≤20 years	WPW syndrome	—	Single AP	—	Excluded	EPS with successful RF ablation	Sensitivity and accuracy of septal AP localization
Li et al. 2019 [22]	Retrospective derivation	Yes	Taiwan	Multicenter	180	Stepwise 12‐lead ECG‐based algorithm	Delta‐wave polarity QRS transition	Pediatric ≤18 years	WPW syndrome	NSR	Single AP	5 half‐lives before ablation	Excluded	EPS with successful RF ablation	This simple, novel algorithm could differentiate left from right AP and septal from free wall AP in pediatric patients. The overall accuracy was 92.3%
Pambrun et al. 2018 [25]	Retrospective derivation + prospective validation	Yes	France + Portugal	Multicenter	207	Stepwise 12‐lead ECG‐based algorithm	Delta‐wave polarity QRS transition	Adults ≥18 years	WPW syndrome	NSR	Single AP	5 half‐lives before ablation	Excluded	EPS with successful RF ablation	An algorithm based on maximal pre‐excitation allows accurate and reproducible localization of manifest APs.
Dung et al. 2018	Retrospective derivation + prospective validation	—	Vietnam	Single‐center	106	Stepwise 12‐lead ECG‐based algorithm	Delta‐wave polarity QRS polarity QRS transition	Adults ≥18 years	WPW syndrome	NSR	Single AP	—	Excluded	EPS with successful RF ablation	12‐lead ECG parameters in typical WPW syndrome are strongly correlated to the septal AP localization, which can be used to develop a new ECG algorithm to localize septal APs with high accuracy.
Taguchi et al. 2013 [28]	Retrospective derivation + prospective validation	—	Japan	Single‐center	144	Stepwise 12‐lead ECG‐based algorithm	QRS transition	Adults ≥18 years	WPW syndrome	NSR	Single AP	5 half‐lives before the study	Excluded	EPS with successful RF ablation	This ECG algorithm provides a simple and accurate way to identify the AP localization.
Fitzpatrick et al. 1994 [10]	Retrospective study	—	USA	Single‐center	180	Stepwise discriminant analysis of pre‐excited ECG features	Delta‐wave polarity QRS polarity QRS transition	Adults ≥18 years	WPW syndrome	NSR	Single AP	—	Excluded	EPS with successful RF ablation	Using the algorithm derived, a right‐sided acces‐sory pathway can be reliably distinguished from one that is left‐sided, right free wall from right septa], right anterelateral from posterolateral and wateroseptal front other right septa] pathways. Left anterolateral pathways can he distinguished from left posterior pathways and left posterolateral pathways from left posteroseptal pathways.
Boersma et al. 2003 [9]	Retrospective diagnostic study	Yes	Spain + The Netherlands	Two centers	157	Stepwise 12‐lead ECG‐based algorithm	QRS polarity	Pediatric <18 years	WPW syndrome	NSR	Single AP	Day of ablation	Excluded	EPS with successful RF ablation	The existing QRS polarity algorithm used to accurately predict AP location in adults was found to be inadequate in children. Only five regions with a similar ECG pattern could be determined with reasonable sensitivity and specificity and overall accuracy of 90%.
Arruda et al. 2007 [8]	Retrospective derivation + prospective validation	—	USA	Single‐center	256	Arruda algorithm	Delta‐wave polarity	Both pediatric & adult	WPW syndrome	NSR	Single AP	—	Included	EPS with successful RF ablation	accurate localization of AP in 10 anatomic sites
Iturralde‐Torres et al. 1996 [19]	Cross‐sectional + retrospective	Yes	Mexico	Single‐center	102	Stepwise 12‐lead ECG‐based algorithm	QRS polarity	Both pediatric & adult	WPW syndrome	NSR	Single	5 half‐lives before the study	—	EPS with successful RF ablation	accurate localization of AP in 5 regions
D'AVILA et al. 1995 [17]	Cross‐sectional + retrospective	Yes	Belgium, Spain + Brazil	Multicenter	140	Stepwise 12‐lead ECG‐based algorithm	QRS polarity	—	WPW syndrome	NSR	Single	5 half‐lives before the study (Except amiodarone)	—	EPS with successful RF ablation	accurate localization of AP in 8 regions
Chiang et al. 1995 [11]	Retrospective derivation + prospective validation	Yes	Taiwan	Multicenter	369	Stepwise 12‐lead ECG‐based algorithm	Delta‐wave polarity QRS transition	Adults ≥18 years	WPW syndrome	NSR	Single	5 half‐lives before the study	—	EPS with successful RF ablation	accurate localization of AP in 13 regions
Xie et al. 1994 [27]	Retrospective derivation + prospective validation	Yes	UK	Single‐center	106	St. George's algorithm	QRS polarity	Both pediatric & adult	WPW syndrome	NSR	Single	—	—	EPS with successful RF ablation	accurate localization of AP. in 9 regions

Abbreviations: AP, accessory pathway; AVRT, atrioventricular reentrant tachycardia; ECG, electrocardiogram; EPM, electrocardiographic pre‐excitation mapping; EPS, electrophysiological study; No., number; NSR, normal sinus rhythm; RF, radiofrequency; UK, United Kingdom; USA, United States of America; WPW, Wolff–Parkinson–White.

### 2.5 Statistical Analysis

Statistical analyses were performed as a diagnostic test accuracy meta‐analysis to compare 12‐lead ECG algorithms for accessory pathway localization. Each ECG algorithm was considered an index test, and an electrophysiology study with successful catheter ablation was used as the reference standard. For each study, diagnostic data were extracted or reconstructed as true positives, false positives, false negatives, and true negatives if sufficient information was reported. Overall localization accuracy was defined as the proportion of patients in whom the ECG algorithm correctly predicted the accessory pathway site confirmed by electrophysiological mapping and ablation. For regional analyses, accessory pathway locations were harmonized into anatomical categories and evaluated using a one‐versus‐rest framework.

Pooled estimates of overall accuracy, sensitivity, specificity, and diagnostic odds ratio were calculated using random‐effects models. Heterogeneity was assessed using Cochran's Q test and the I^2^ statistic. Summary receiver operating characteristic analysis was performed using the Reitsma bivariate model to jointly summarize sensitivity and false‐positive rate while accounting for their paired nature [15]. Leave‐one‐out sensitivity analyses were performed by sequentially excluding each study and recalculating pooled estimates. A two‐sided p‐value <0.05 was considered statistically significant.

## 3 Results

### 3.1 Study Selection and Characteristics

The systematic literature search and screening process yielded 17 diagnostic accuracy studies [8, 9, 10, 11, 16, 17, 18, 19, 20, 21, 22, 23, 24, 25, 26, 27, 28], published between 1993 and 2025, comprising a pooled cohort of 3,159 patients with manifest WPW syndrome or ventricular pre‐excitation. The detailed study selection process is presented in the PRISMA flow diagram (Figure 1). The selected studies represented a geographically diverse population across Europe, Asia, and the Americas, and were conducted in both single‐center (*n* = 9) and multicenter (*n* = 8) settings. A summary of these characteristics is provided in Table 1. Demographic representation spanned all age groups, with eight studies exclusively evaluating adult patients, three focusing entirely on pediatric populations, and six including mixed age demographics. Detailed baseline clinical and demographic characteristics of the pooled patient cohort are summarized in Table 1. Uniformly across all included studies, the reference standard for confirming true accessory pathway localization was an invasive electrophysiological study (EPS) with successful catheter ablation.

**FIGURE 1:**
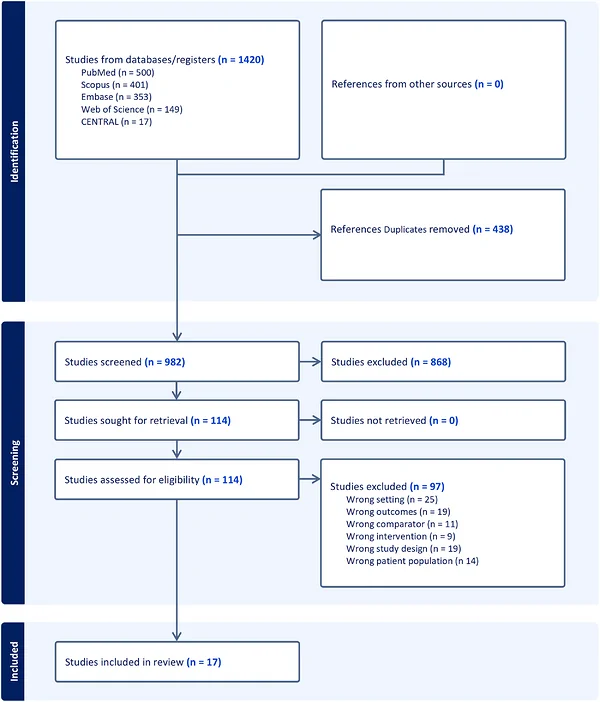
PRISMA flow diagram for the systematic search and study selection process. [Colour figure can be viewed at wileyonlinelibrary.com]

### 3.2 Quality Assessment

Quality assessment using the QUADAS‐2 tool indicated a generally low risk of bias across the included studies. Fifteen of the 17 studies were rated as low risk in all four risk‐of‐bias domains. The remaining two studies each had one unclear judgment: Baek et al. (2020) in the index‐test domain because blinding of ECG interpretation was insufficiently reported, and Raharjo et al. (2022) in the flow‐and‐timing domain because relevant timing or participant‐flow details were unclear. No study received a high‐risk judgment in any domain. Patient selection and the reference standard were rated as low risk in all studies, while applicability concerns were uniformly low across all three domains. Overall, major methodological concerns were uncommon, although these isolated reporting limitations should be considered when interpreting the pooled findings (Figure 2).

**FIGURE 2:**
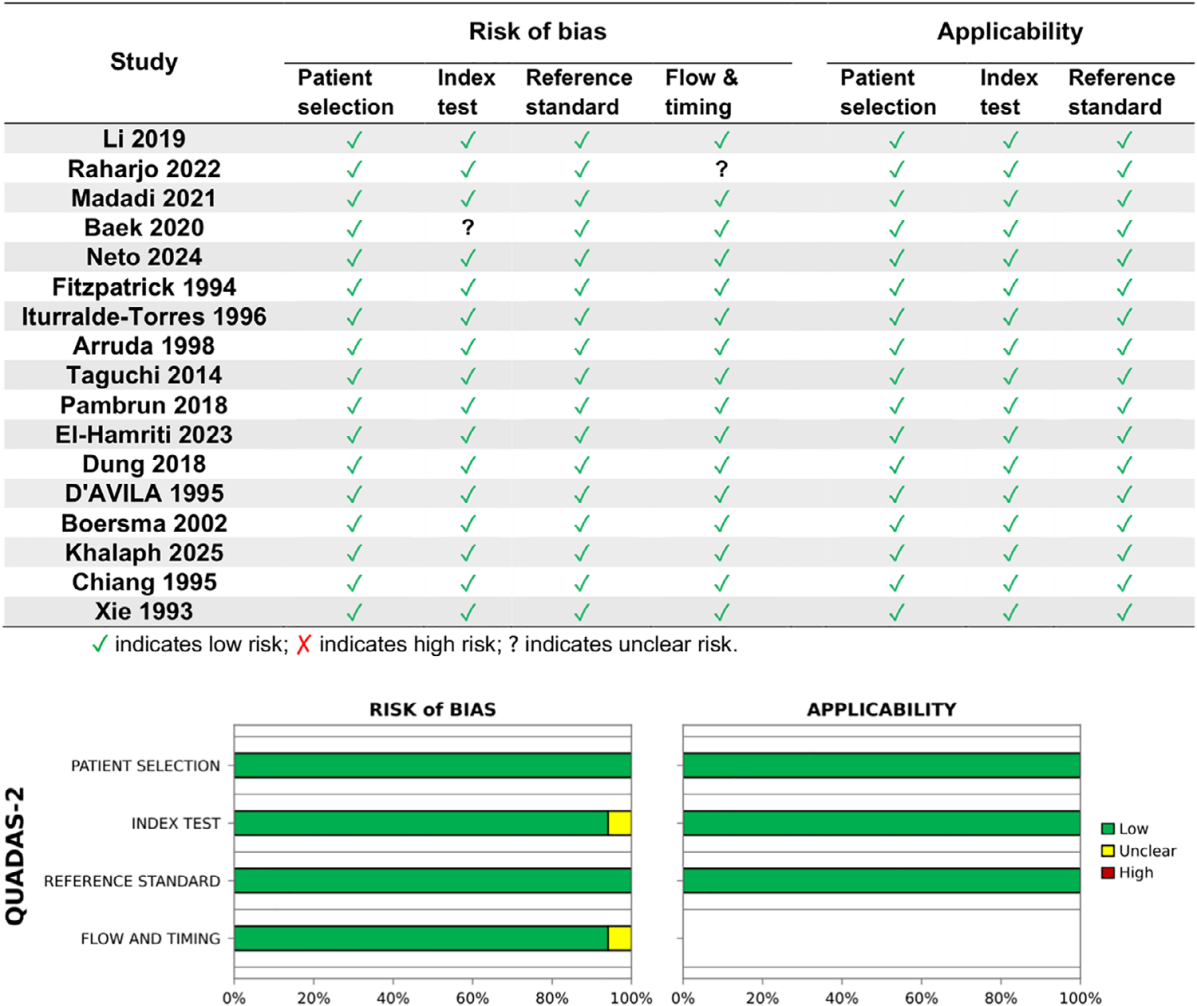
Risk of bias and applicability concerns of the included studies assessed using the QUADAS‐2 tool. The upper panel presents study‐level judgments, and the lower panel summarizes judgments across the QUADAS‐2 domains. [Colour figure can be viewed at wileyonlinelibrary.com]

### 3.3 Overall Diagnostic Accuracy and Discriminative Performance

In the primary analysis of diagnostic performance, the pooled overall accuracy of 12‐lead ECG algorithms for localizing accessory pathways was 0.91 (95% CI, 0.85‐0.94) using a random‐effects model. Substantial between‐study and between‐algorithm heterogeneity was observed (I^2^ = 91.4; *p* < 0.0001) (Figure 3a).

**FIGURE 3:**
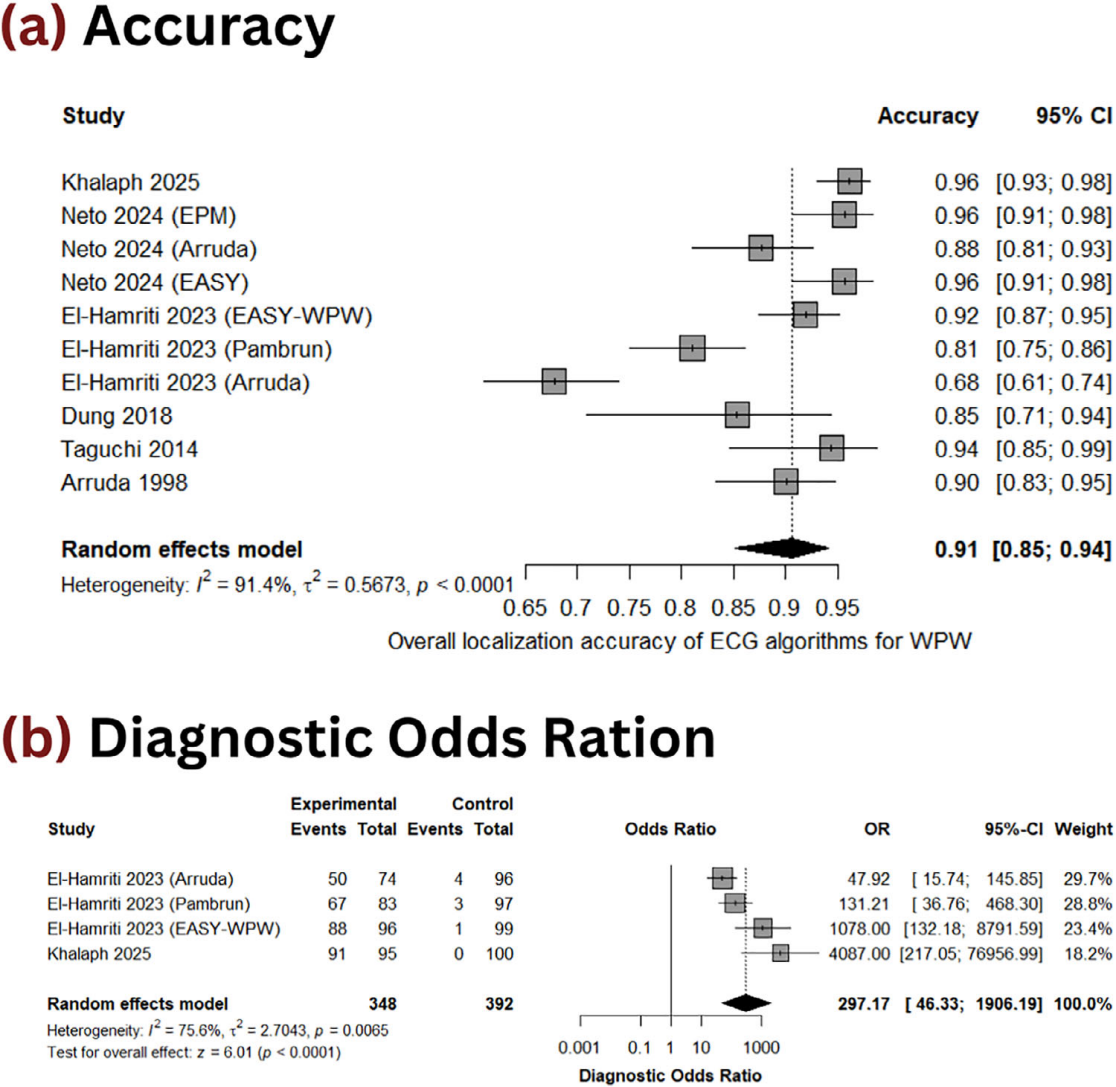
Forest plots showing (a) pooled overall localization accuracy and (b) pooled diagnostic odds ratio of 12‐lead ECG algorithms for accessory pathway localization using random‐effects models. CI, confidence interval; ECG, electrocardiogram. [Colour figure can be viewed at wileyonlinelibrary.com]

The pooled diagnostic odds ratio (DOR), evaluating the overall discriminative effectiveness of the algorithms, was 297.17 (95% CI, 46.33–1906.19; *p* < 0.0001). This point estimate indicates strong diagnostic utility, though accompanied by significant heterogeneity (I^2^ = 75.6%; *p* = 0.0065) (Figure 3b). Furthermore, summary receiver operating characteristic (SROC) analysis using the Reitsma model demonstrated robust overall performance, with the joint distribution of sensitivity and false‐positive rates clustering in the upper‐left quadrant of the ROC space, reflecting a high overall area under the curve (Figure 4).

**FIGURE 4:**
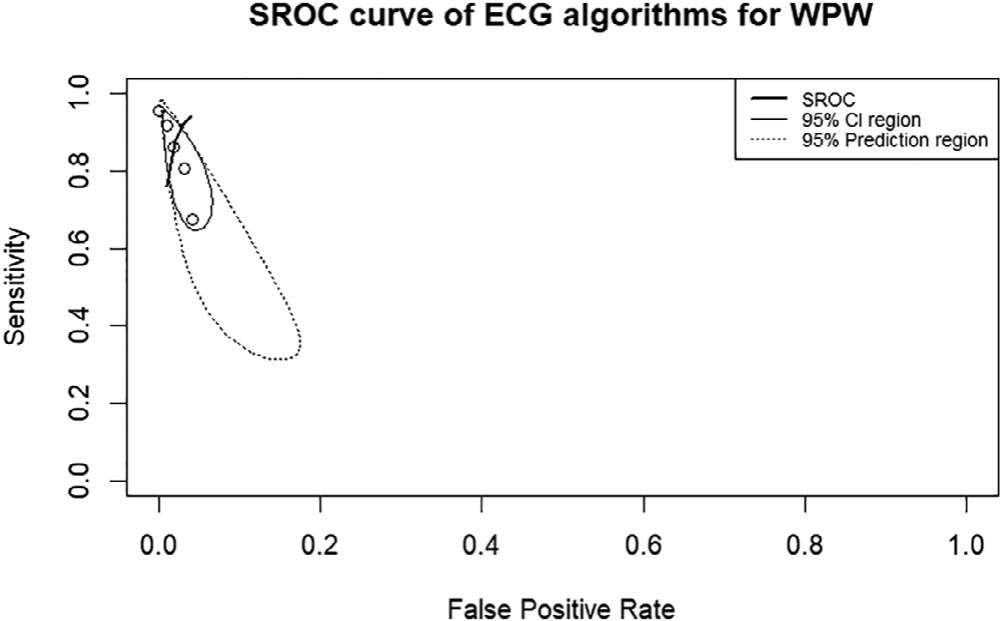
Summary receiver operating characteristic curve of 12‐lead ECG algorithms for accessory pathway localization using the Reitsma bivariate model, with 95% confidence and prediction regions.

### 3.4 Sensitivity and Specificity

When evaluating specific diagnostic parameters, the algorithms demonstrated a pooled sensitivity of 0.87 (95% CI, 0.73‐0.94). Consistent with the primary accuracy findings, sensitivity exhibited high heterogeneity (I^2^ = 88.3%; *p* < 0.0001), with individual algorithm estimates ranging from 0.68 to 0.96 (Figure 5b).

**FIGURE 5:**
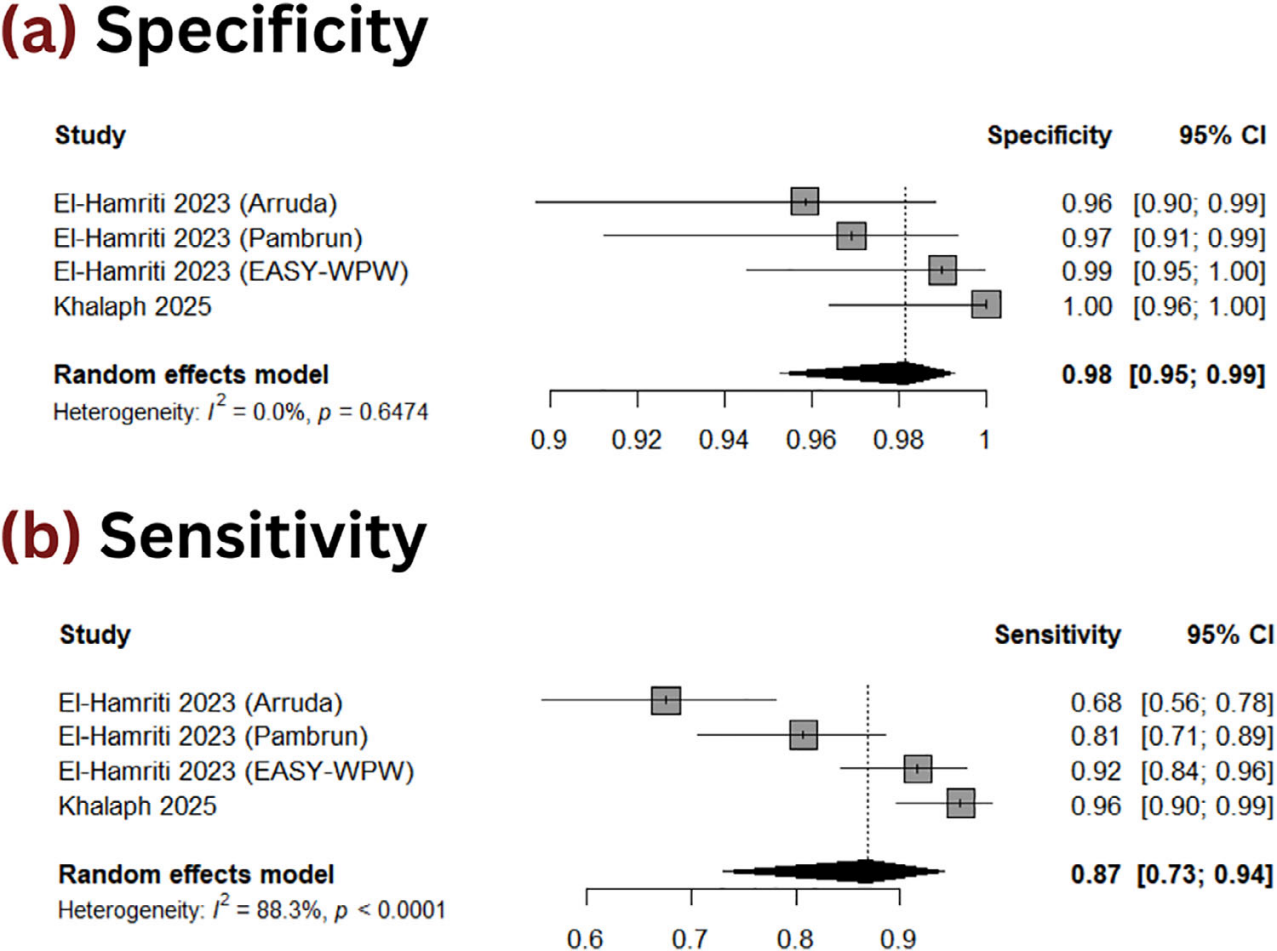
Forest plots showing pooled (a) specificity and (b) sensitivity of 12‐lead ECG algorithms for accessory pathway localization using random‐effects models. CI, confidence interval; ECG, electrocardiogram. [Colour figure can be viewed at wileyonlinelibrary.com]

In contrast, the ECG algorithms demonstrated a highly homogenous and robust pooled specificity of 0.98 (95% CI, 0.95‐0.99) (Figure 5a). Heterogeneity was absent for this parameter (I^2^ = 0.0%; *p* = 0.6474), indicating that although the algorithms varied in their ability to correctly identify the exact pathway location, they consistently yielded a very low rate of false‐positive localization for any given anatomical region.

### 3.5 Sensitivity Analysis

The primary diagnostic outcomes proved highly robust, indicating that the overall findings were not disproportionately driven by outlier data or a single heavily weighted investigation. Specifically, when evaluating overall localization accuracy, the recalculated pooled proportions remained remarkably stable, fluctuating only marginally between 0.90 and 0.92 (Figure S1). Similarly, the LOO analysis for sensitivity demonstrated consistent pooled estimates ranging from 0.82 to 0.91 (Figure S2). Ultimately, these sensitivity analyses confirm the high reliability of the pooled diagnostic results, demonstrating that the strong aggregate performance of these ECG algorithms is a consistent finding across the literature rather than an effect of a few influential studies.

### 3.6 Anatomical Patterns of Misclassification

Only a subset of studies reported the anatomical destination of incorrect predictions. Among the available reports, many errors involved adjacent or anatomically related regions, although occasional more distant assignments were also described. In Chiang et al., 11 of 13 incorrect predictions (84.6%) were adjacent to the confirmed site [11]. Fitzpatrick et al. reported that 4 of 17 right posteroseptal pathways were classified as midseptal and 2 of 7 midseptal pathways were classified as right posteroseptal; isolated misclassifications between right septal and right free‐wall locations were also observed [10]. When an adult algorithm was applied to a pediatric cohort, Boersma et al. found considerable difficulty differentiating septal and right‐sided pathways [9]. Taguchi et al. provided predicted‐versus‐confirmed cross‐tabulations for both the derivation and validation cohorts, but their classification system combined contiguous locations into five composite regions, limiting assessment of exact subregional error destinations [29]. Taken together, these findings suggest that many reported errors occurred near the confirmed site, but they do not establish a consistent adjacent‐region error pattern across all algorithms. A pooled wrong‐region analysis was therefore not considered appropriate because studies used non‐equivalent anatomical classifications and most did not report complete multicategory confusion matrices. Study‐level findings are summarized in Table S3.

## 4 Discussion

In this systematic review and meta‐analysis of 17 diagnostic accuracy studies including patients with manifest WPW syndrome or ventricular pre‐excitation, 12‐lead ECG algorithms showed strong overall performance for localization of accessory pathways. The pooled overall localization accuracy was 0.91, with a pooled sensitivity of 0.87 and a pooled specificity of 0.98. The diagnostic odds ratio was also high, supporting strong overall discriminative ability. Clinically, these findings suggest that surface ECG algorithms can provide a useful pre‐procedural estimate of pathway location before EPS and catheter ablation. Biologically, this is plausible because the delta wave and early QRS morphology reflect the earliest ventricular activation site produced by antegrade conduction over the accessory pathway. A recent computational electrophysiology study also showed that accessory pathway location and timing can substantially influence 12‐lead ECG morphology, supporting the mechanistic basis of ECG‐based localization [30].

The high overall accuracy observed in our analysis supports the value of ECG‐based localization as a practical first step in procedural planning. Correct prediction of whether the pathway is left‐sided, right‐sided, septal, or related to the coronary sinus may influence vascular access, mapping strategy, need for transseptal or retro‐aortic approach, anticipated procedural difficulty, and pre‐procedural counseling. This is clinically relevant because accurate prediction of accessory pathway location has been linked to planning of ablation approach, risk prediction, and procedural outcome [20]. Left‐sided pathways may require transseptal or retro‐aortic access, whereas septal and para‐Hisian pathways require careful mapping because of their proximity to the normal conduction system [30]. The pooled sensitivity of 0.87 indicates that most true pathway locations were correctly identified by ECG algorithms, but a clinically meaningful minority may still be missed or assigned to an adjacent region. This finding is consistent with the known complexity of accessory pathway localization. Septal pathways, para‐Hisian pathways, multiple adjacent anatomical zones, incomplete pre‐excitation, and variation in ECG interpretation can all reduce sensitivity [31, 32]. The computational study by Gillette et al. specifically demonstrated failure mechanisms of ECG algorithms in pathways inserting on the right and left sides of the septum, which may explain part of the residual false‐negative rate in our pooled estimate [30]. Clinically, this means that a negative or non‐matching ECG prediction should not be used to exclude a pathway region during EPS. Instead, the ECG algorithm should be interpreted as a guide that can narrow the initial mapping strategy while preserving full invasive confirmation.

The pooled specificity of 0.98 was the strongest diagnostic parameter in our analysis. This suggests that when an ECG algorithm identifies a given anatomical region, the probability of a false‐positive assignment is low. Clinically, high specificity is useful because it may help operators prepare for region‐specific risks. For example, a highly specific septal prediction may alert the operator to possible proximity to the AV node and His bundle, while a highly specific left‐sided prediction may support early planning for left‐sided access [31, 32]. However, this high specificity should be interpreted carefully. In diagnostic accuracy studies that evaluate regional localization, specificity can be high because each target region is commonly assessed against several non‐target regions. Therefore, high specificity does not necessarily indicate perfect exact localization within closely adjacent subregions, especially when broad regional or one‐versus‐rest classification is used instead of exact site prediction [29, 33, 34, 35]. Our findings appear more favorable than some pediatric validation data. Kurath‐Koller et al. evaluated 12 algorithms in pediatric patients with WPW and found that the highest exact localization accuracy was only 58% for the D'Avila and Boersma algorithms, while median accuracy for left versus right‐sided localization was 74% and for septal pathways was 68% [36]. This difference may be explained by several factors. First, pediatric ECG morphology differs from adult ECG morphology, and many classic algorithms were developed mainly in adult cohorts [37]. Second, exact localization into multiple anatomical sites is more demanding than broad regional classification. Third, some studies used ECGs with full pre‐excitation, while others used resting ECGs with variable degrees of fusion, which may reduce algorithm performance [31]. Therefore, our pooled estimate should be interpreted as evidence of strong overall diagnostic utility across studies, but not as proof that every algorithm performs equally well in every age group or anatomical subgroup.

Compared with previous literature, our study addresses a distinct evidence gap. A recent review systematically analyzed classical and recent rule‐based ECG algorithms as well as deep learning models for accessory pathway localization, emphasizing their value for pre‐interventional planning [38]. However, previous quantitative meta‐analyses in WPW have mainly focused on treatment outcomes rather than pooled diagnostic performance of ECG localization algorithms. For example, a meta‐analysis of radiofrequency ablation in WPW reported a pooled success rate of 94.1%, recurrence rate of 6.2%, and complication rate of 1% [39]. These treatment outcomes make accurate localization clinically important, because ablation is highly effective when the pathway is correctly mapped and safely targeted.

Our findings also complement prior meta‐analyses evaluating other non‐invasive strategies in WPW. A diagnostic meta‐analysis of exercise testing for detecting high‐risk accessory pathways reported high sensitivity of 92.7% but low specificity of 28.1% [40]. This differs from our findings because exercise testing assesses conduction risk rather than anatomical localization. Risk stratification depends on loss or persistence of pre‐excitation and accessory pathway refractory properties, whereas localization depends on spatial ECG morphology. Thus, both approaches answer different clinical questions. Exercise testing may help identify lower‐risk conduction behavior, while localization algorithms may help guide procedural planning once EPS or ablation is being considered.

The substantial heterogeneity in overall accuracy and sensitivity is important. It likely reflects differences in study design, algorithm complexity, patient age, pathway distribution, anatomical classification, blinding, ECG acquisition, and reference standard definitions. Older algorithms often relied on simpler ECG rules based on QRS polarity, delta‐wave morphology, or R/S ratios, whereas newer algorithms incorporate structured anatomical decision trees and maximal pre‐excitation criteria [11, 21, 25, 28, 37, 41]. Heterogeneity may also be increased by combining exact localization outcomes with broader regional discrimination in some datasets. Despite this heterogeneity, leave‐one‐out sensitivity analysis showed stable pooled estimates, suggesting that the main results were not driven by a single study.

### 4.1 Clinical Implications

Current guidelines emphasize EPS and catheter ablation for symptomatic or high‐risk patients rather than relying on ECG localization alone. The 2019 ESC SVT guidelines recommend catheter ablation of the accessory pathway in patients with symptomatic recurrent AVRT and recommend EPS with isoprenaline for risk stratification in selected asymptomatic individuals, including those with high‐risk occupations or competitive athletics [41]. The same guideline recommends ablation in asymptomatic patients when EPS identifies high‐risk properties such as SPERRI ≤ 250 ms, accessory pathway ERP ≤ 250 ms, multiple pathways, or inducible pathway‐mediated tachycardia. The 2015 ACC/AHA/HRS guideline summary similarly states that patients with intermittent pre‐excitation or abrupt loss of pre‐excitation during exercise are generally considered low risk, while others may undergo EP testing, and ablation should be performed when high‐risk features are found [42]. Our findings do not change these treatment recommendations. ECG algorithms cannot replace EPS, invasive mapping, or clinical risk stratification. However, they may add value before the procedure by improving anatomical anticipation, operator planning, patient counseling, and selection of mapping strategy. This is especially relevant because septal ablation carries different safety considerations than free‐wall ablation. A meta‐analysis of septal accessory pathway ablation showed that radiofrequency ablation had higher efficacy than cryoablation, but with a higher risk of AV block, particularly in mid‐septal and para‐Hisian regions [43]. Therefore, a reliable pre‐procedural ECG estimate may help prepare for these anatomical risks while keeping EPS as the final reference standard.

This meta‐analysis quantifies the diagnostic classification performance of ECG localization algorithms but cannot determine whether their use improves procedural workflow or clinical outcomes. The included studies did not provide comparative evidence regarding mapping time, procedure duration, fluoroscopy exposure, ablation success, recurrence, complications, or patient‐reported outcomes. Therefore, any downstream workflow or clinical benefit remains plausible but unproven and should be evaluated in prospective comparative studies.

#### 4.1.1 Strengths, Limitations, and Recommendations

This study has several strengths. It includes a broad set of ECG algorithms across adult, pediatric, single‐center, and multicenter cohorts. The reference standard was consistently based on EPS and successful ablation site. We also assessed multiple diagnostic domains, including overall accuracy, sensitivity, specificity, diagnostic odds ratio, SROC performance, and leave‐one‐out sensitivity.

Several limitations should be acknowledged. First, heterogeneity was substantial for overall accuracy and sensitivity. Second, the included studies differed in anatomical classification, with some reporting exact pathway localization and others reporting broader regional or binary discrimination. Third, not all studies reported complete 2×2 data, limiting more detailed subgroup and comparative analyses. Fourth, several algorithms were assessed in derivation cohorts or selected validation samples, which may overestimate performance. Fifth, pediatric and adult data were pooled despite differences in ECG morphology and pathway distribution. Finally, the current evidence is mostly based on diagnostic performance rather than patient‐centered procedural outcomes such as procedure time, fluoroscopy exposure, ablation success, recurrence, and complications. We were also unable to quantify which anatomical regions were most commonly assigned when an algorithm failed. The limited available evidence suggests that misclassification generally occurred between neighboring regions; however, this finding should be interpreted cautiously. Studies differed in their anatomical definitions, degree of pre‐excitation and anterograde fusion, accessory pathway orientation, and patient age, and most did not report complete predicted‐versus‐confirmed cross‐tabulations. We therefore summarized these error patterns descriptively rather than pooling non‐equivalent data. Future validation studies should use standardized anatomical classifications and report complete multicategory confusion matrices.

Future studies should use prospective multicenter designs, blinded ECG interpretation, standardized anatomical categories, complete 2×2 reporting, and external validation across age groups. Studies should also compare ECG algorithms head‐to‐head and evaluate whether algorithm‐guided planning improves procedural efficiency, radiation exposure, ablation success, and safety. Integration of rule‐based algorithms with machine learning or patient‐specific computational modeling may also improve localization in difficult regions such as septal and para‐Hisian pathways. Additionally, future studies should move beyond diagnostic accuracy and prospectively compare algorithm‐guided planning with standard clinical practice. These studies should determine whether ECG localization meaningfully reduces mapping time, procedure duration, fluoroscopy exposure, complications, or recurrence and improves patient‐reported outcomes.

## 5 Conclusion

In patients with manifest WPW syndrome or ventricular pre‐excitation, 12‐lead ECG algorithms show strong overall diagnostic performance for accessory pathway localization, with particularly high specificity and stable pooled estimates. These algorithms are clinically useful as non‐invasive pre‐procedural planning tools, but they should not replace EPS or invasive mapping. Their greatest value may be in anticipating pathway region, procedural approach, and anatomical risk before ablation.

## Funding

No external or institutional funding was received for the conduct of this study.

## Disclosure

Christian Sohns received research support and lecture fees from Medtronic, Abbott, Boston Scientific, and J&J MedTec; is a consultant for Medtronic, Boston Scientific, and J&J MedTec. Philipp Sommer is Member of Advisory Board for Abbott, Boston Scientific, J&J MedTech and Medtronic. All other author's state: None

## Ethics Statement

As this study was based exclusively on previously published data, ethical approval was not required.

## Consent

Informed consent was not required.

## Conflicts of Interest

The authors declare no conflicts of interest.

## Supporting information




**Supporting File**: pace70387‐sup‐0001‐SuppMat.pdf

## Data Availability

All data underlying the findings of this study were obtained from publicly accessible published studies and online databases.
