# Supplementary material for: Diagnostic Accuracy of 12‐Lead ECG Algorithms for Accessory Pathway Localization in WPW: A Meta‐Analysis
Source: Pacing Clin Electrophysiol. 2026 Aug 11;49(11):1748–61. doi: 10.1111/pace.70387 (PMC13739752; doi:10.1111/pace.70387)

Index.

Supplementary Content:		
**a. Tables:**		**Page**
1	**Supplementary Table 1.** PRISMA-DTA reporting checklist.	3
2	**Supplementary Table 2.** Electronic database search strategies.	5
3	**Supplementary Table 3.** Study-reported anatomical destinations of incorrect ECG algorithm predictions.	7
**b. Figures:**		**Page**
1	**Supplementary Fig. 1.** Leave-one-out analysis of pooled overall localization accuracy.	8
2	**Supplementary Fig. 2.** Leave-one-out analysis of pooled sensitivity.	9

Supplementary Table 1. PRISMA-DTA reporting checklist.

Section/topic	#	PRISMA-DTA Checklist Item	Reported on page #
**TITLE / ABSTRACT**			
Title	1	Identify the report as a systematic review (+/- meta-analysis) of diagnostic test accuracy (DTA) studies.	1
Abstract	2	Abstract: See PRISMA-DTA for abstracts.	3-4
**INTRODUCTION**			
Rationale	3	Describe the rationale for the review in the context of what is already known.	5
Clinical role of index test	D1	State the scientific and clinical background, including the intended use and clinical role of the index test, and if applicable, the rationale for minimally acceptable test accuracy (or minimum difference in accuracy for comparative design).	5; 15-16
Objectives	4	Provide an explicit statement of question(s) being addressed in terms of participants, index test(s), and target condition(s).	3; 5
**METHODS**			
Protocol and registration	5	Indicate if a review protocol exists, if and where it can be accessed (e.g., Web address), and, if available, provide registration information including registration number.	6
Eligibility criteria	6	Specify study characteristics (participants, setting, index test(s), reference standard(s), target condition(s), and study design) and report characteristics (e.g., years considered, language, publication status) used as criteria for eligibility, giving rationale.	6-7
Information sources	7	Describe all information sources (e.g., databases with dates of coverage, contact with study authors to identify additional studies) in the search and date last searched.	6
Search	8	Present full search strategies for all electronic databases and other sources searched, including any limits used, such that they could be repeated.	6; Supplementary Search Strategy, pp. 1-3
Study selection	9	State the process for selecting studies (i.e., screening, eligibility, included in systematic review, and, if applicable, included in the meta-analysis).	6-7
Data collection process	10	Describe method of data extraction from reports (e.g., piloted forms, independently, in duplicate) and any processes for obtaining and confirming data from investigators.	7
Definitions for data extraction	11	Provide definitions used in data extraction and classifications of target condition(s), index test(s), reference standard(s) and other characteristics (e.g. study design, clinical setting).	7-8
Risk of bias and applicability	12	Describe methods used for assessing risk of bias in individual studies and concerns regarding the applicability to the review question.	8; 9-10
Diagnostic accuracy measures	13	State the principal diagnostic accuracy measure(s) reported (e.g. sensitivity, specificity) and state the unit of assessment (e.g. per-patient, per-lesion).	7-8
Synthesis of results	14	Describe methods of handling data, combining results of studies and describing variability between studies. This could include, but is not limited to: a) handling of multiple definitions of target condition. b) handling of multiple thresholds of test positivity, c) handling multiple index test readers, d) handling of indeterminate test results, e) grouping and comparing tests, f) handling of different reference standards	8
Meta-analysis	D2	Report the statistical methods used for meta-analyses, if performed.	8
Additional analyses	16	Describe methods of additional analyses (e.g., sensitivity or subgroup analyses, meta-regression), if done, indicating which were pre-specified.	8; 11
**RESULTS**			
Study selection	17	Provide numbers of studies screened, assessed for eligibility, included in the review (and included in meta-analysis, if applicable) with reasons for exclusions at each stage, ideally with a flow diagram.	9; Figure 1
Study characteristics	18	For each included study provide citations and present key characteristics including: a) participant characteristics (presentation, prior testing), b) clinical setting, c) study design, d) target condition definition, e) index test, f) reference standard, g) sample size, h) funding sources	9; Tables 1-2
Risk of bias and applicability	19	Present evaluation of risk of bias and concerns regarding applicability for each study.	9-10
Results of individual studies	20	For each analysis in each study (e.g. unique combination of index test, reference standard, and positivity threshold) report 2x2 data (TP, FP, FN, TN) with estimates of diagnostic accuracy and confidence intervals, ideally with a forest or receiver operator characteristic (ROC) plot.	10-11; Figures 2-6
Synthesis of results	21	Describe test accuracy, including variability; if meta-analysis was done, include results and confidence intervals.	10-11

Section/topic	#	PRISMA-DTA Checklist Item	Reported on page #
Additional analysis	23	Give results of additional analyses, if done (e.g., sensitivity or subgroup analyses, meta-regression; analysis of index test: failure rates, proportion of inconclusive results, adverse events).	11
**DISCUSSION**			
Summary of evidence	24	Summarize the main findings including the strength of evidence.	12-15
Limitations	25	Discuss limitations from included studies (e.g. risk of bias and concerns regarding applicability) and from the review process (e.g. incomplete retrieval of identified research).	16
Conclusions	26	Provide a general interpretation of the results in the context of other evidence. Discuss implications for future research and clinical practice (e.g. the intended use and clinical role of the index test).	16-17
**FUNDING**			
Funding	27	For the systematic review, describe the sources of funding and other support and the role of the funders.	18

Abbreviations: DTA, diagnostic test accuracy; PRISMA-DTA, Preferred Reporting Items for a Systematic Review and Meta-analysis of Diagnostic Test Accuracy Studies.

Supplementary Table 2. Electronic database search strategies.

Database	Field/filter	Access date	Search strategy	No. of results
PubMed	All fields	12 December 2025	("wolff-parkinson-white" OR "wolff parkinson white" OR wpw OR preexcitation OR "pre-excitation" OR "accessory pathway" OR "accessory pathways" OR "accessory conduction pathway" OR "accessory atrioventricular pathway") AND (ecg OR electrocardiogram OR "12-lead" OR "12 lead" OR "surface ecg") AND (algorithm* OR criteria OR "scoring system" OR score* OR "prediction rule" OR predict* OR localization OR localisation OR localiz* OR localis* OR mapping) AND ("diagnostic accuracy" OR sensitivity OR specificity OR "predictive value" OR "predictive values" OR accuracy OR validity)	500
Cochrane CENTRAL	All fields	12 December 2025	("wolff-parkinson-white" OR "wolff parkinson white" OR wpw OR preexcitation OR "pre-excitation" OR "accessory pathway" OR "accessory pathways" OR "accessory conduction pathway" OR "accessory atrioventricular pathway") AND (ecg OR electrocardiogram OR "12-lead" OR "12 lead" OR "surface ecg") AND (algorithm* OR criteria OR "scoring system" OR score* OR "prediction rule" OR predict* OR localization OR localisation OR localiz* OR localis* OR mapping) AND ("diagnostic accuracy" OR sensitivity OR specificity OR "predictive value" OR "predictive values" OR accuracy OR validity)	17
Scopus	Title, abstract, and keywords	12 December 2025	("wolff-parkinson-white" OR "wolff parkinson white" OR wpw OR preexcitation OR "pre-excitation" OR "accessory pathway" OR "accessory pathways" OR "accessory conduction pathway" OR "accessory atrioventricular pathway") AND (ecg OR electrocardiogram OR "12-lead" OR "12 lead" OR "surface ecg") AND (algorithm* OR criteria OR "scoring system" OR score* OR "prediction rule" OR predict* OR localization OR localisation OR localiz* OR localis* OR mapping) AND ("diagnostic accuracy" OR sensitivity OR specificity OR "predictive value" OR "predictive values" OR accuracy OR validity)	401
Web of Science	All fields	12 December 2025	("wolff-parkinson-white" OR "wolff parkinson white" OR wpw OR preexcitation OR "pre-excitation" OR "accessory pathway" OR "accessory pathways" OR "accessory conduction pathway" OR "accessory atrioventricular pathway") AND (ecg OR electrocardiogram OR "12-lead" OR "12 lead" OR "surface ecg") AND (algorithm* OR criteria OR "scoring system" OR score* OR "prediction rule" OR predict* OR localization OR localisation OR localiz* OR localis* OR mapping) AND ("diagnostic accuracy" OR sensitivity OR specificity OR "predictive value" OR "predictive values" OR accuracy OR validity)	149

Database	Field/filter	Access date	Search strategy	No. of results
Embase	All fields	12 December 2025	("wolff-parkinson-white" OR "wolff parkinson white" OR wpw OR preexcitation OR "pre-excitation" OR "accessory pathway" OR "accessory pathways" OR "accessory conduction pathway" OR "accessory atrioventricular pathway") AND (ecg OR electrocardiogram OR "12-lead" OR "12 lead" OR "surface ecg") AND (algorithm* OR criteria OR "scoring system" OR score* OR "prediction rule" OR predict* OR localization OR localisation OR localiz* OR localis* OR mapping) AND ("diagnostic accuracy" OR sensitivity OR specificity OR "predictive value" OR "predictive values" OR accuracy OR validity)	353
**Total**				**1,420**

Supplementary Table 3. Study-reported anatomical destinations of incorrect ECG algorithm predictions.

Study	Error-reporting format	Study-reported misclassification pattern	Reason not pooled
Xie et al., 1994	Predicted-versus-actual table in 46 prospectively analyzed patients	The study reported algorithm-specific predicted and confirmed sites for Milstein, Skeberis, and St. George algorithms. Accuracy differed according to whether 4, 7, or 9 sites were used.	Multiple algorithms and non-equivalent 4-, 7-, and 9-site schemes.
Chiang et al., 1995	Narrative description of errors in a prospective cohort	Thirteen of 187 predictions were incorrect; 11 of the 13 errors (84.6%) were to a site adjacent to the confirmed location.	Detailed multicategory cell counts were not provided for all sites.
d'Avila et al., 1995	Narrative list of 12 misclassified pathways	Twelve of 140 pathways (8.5%) were misclassified. Reported errors included left lateral to left posterior; posteroseptal to left posterior or right posteroseptal; right posteroseptal to right lateral or posteroseptal; anteroseptal to right lateral or right posteroseptal; and midseptal to posteroseptal or right posteroseptal.	Eight-region study-specific scheme and narrative rather than a common matrix.
Arruda et al., 1998	Predicted-versus-actual matrix in prospective validation	The algorithm correctly localized 109 of 121 pathways. The published matrix used 10 anatomic sites and included specialized septal and coronary venous categories.	Ten-site scheme was not directly compatible with broader regional schemes.
Fitzpatrick et al., 1994	Pairwise classification matrices and narrative error counts	Reported errors included left posteroseptal classified as right-sided; right anteroseptal versus right lateral/free-wall confusion; four of 17 right posteroseptal pathways classified as midseptal; two of seven midseptal pathways classified as right posteroseptal; and two left posterolateral pathways classified as posteroseptal.	Different pairwise discriminant models rather than one common multicategory matrix.
Boersma et al., 2002	Narrative error pattern for adult algorithm applied to children	The adult algorithm had 53% accuracy, 38% misdiagnosis, and 9% failure to reach a diagnosis. Errors included left lateral to left posteroseptal, left posteroseptal to left posterior, anteroseptal to right lateral, and difficulty separating posteroseptal, right posteroseptal, and right lateral pathways. The pediatric algorithm then grouped pathways into five composite regions.	Adult and pediatric schemes differed, with unclassified cases and composite regions.
Taguchi et al., 2014	Predicted-versus-actual matrices in derivation and validation cohorts	The algorithm correctly localized 82 of 88 pathways in development and 51 of 54 in prospective validation. Errors occurred across five composite regions that deliberately grouped contiguous sites.	Composite five-region scheme obscured exact within-region error destinations.

Abbreviations: AP, accessory pathway; ECG, electrocardiogram; EPS, electrophysiological study.

Supplementary Fig. 1. Leave-one-out analysis of pooled overall localization accuracy.

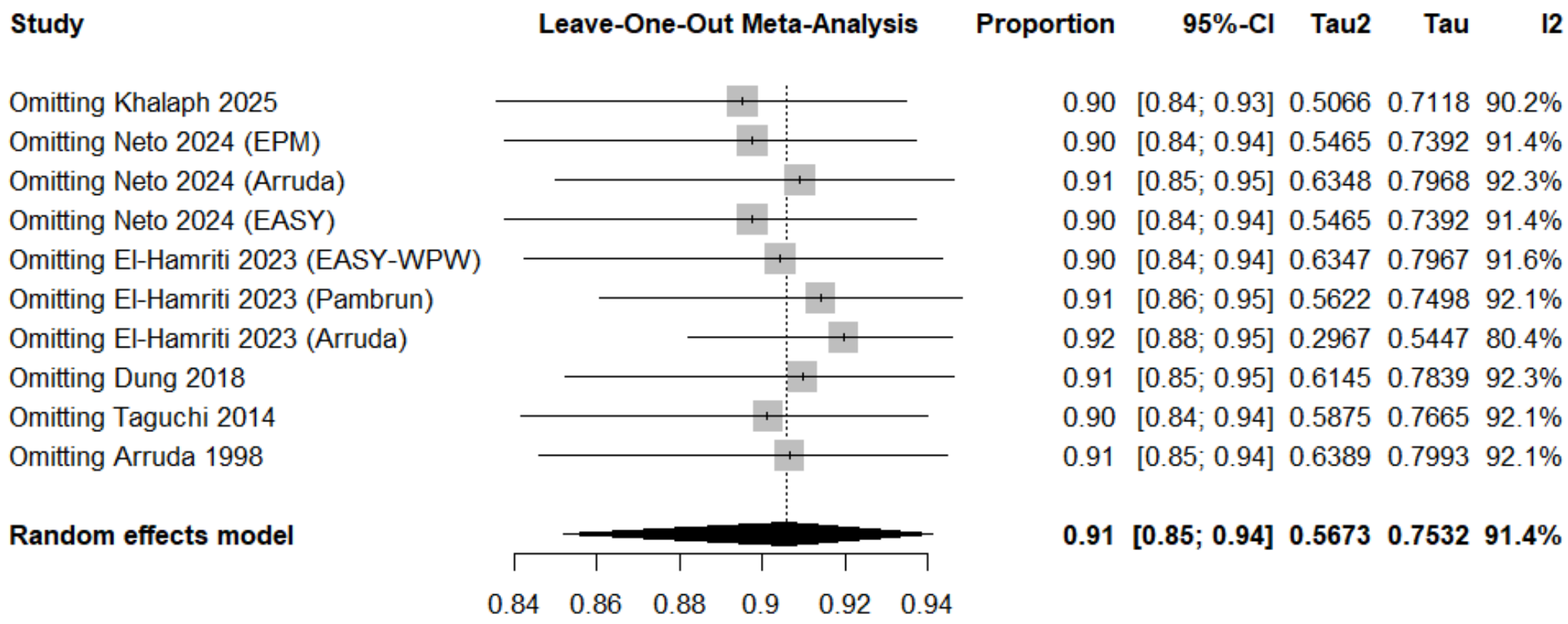

Supplementary Fig. 2. Leave-one-out analysis of pooled sensitivity.

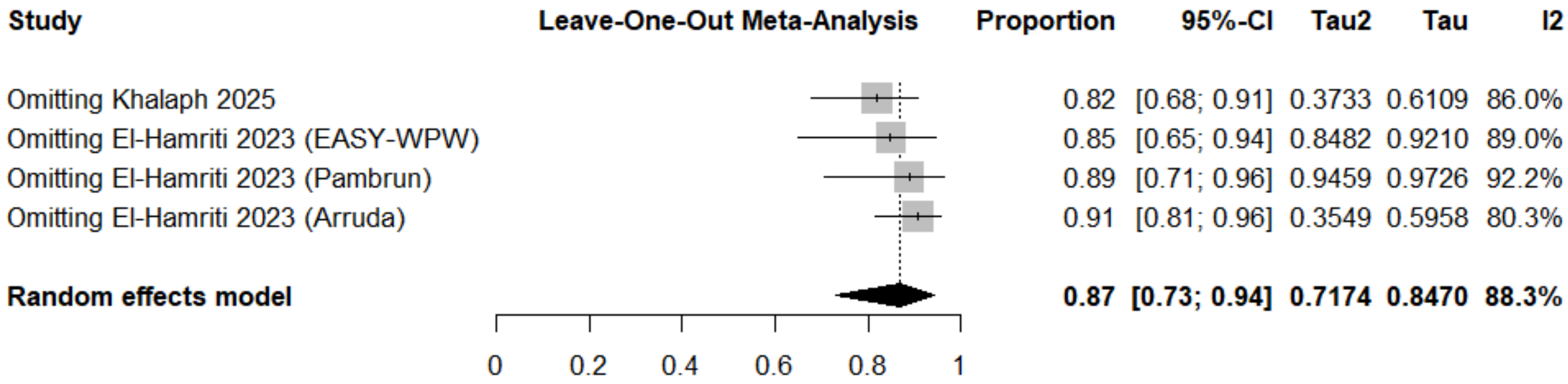

Supplement: Supplementary file 1 [file PACE-49-1748-s001.pdf]
